# Separation of Nickel-Titanium Rotary and Reciprocating Instruments: A Mini-Review of Clinical Studies

**DOI:** 10.2174/1745017901814010864

**Published:** 2018-10-25

**Authors:** Marcelo Santos Coelho, Marcos de Azevêdo Rios, Carlos Eduardo da Silveira Bueno

**Affiliations:** 1 Department of Endodontics, São Leopoldo Mandic Dental Research Center, Campinas, SP, Brazil; 2 Department of Endodontics, State University of Feira de Santana, Feira de Santana, Brazil

**Keywords:** Nickel-titanium, Root canal treatment, Rotary, Reciprocating, Separation, Dentinal defects

## Abstract

**Objective:**

This review aimed to evaluate clinical studies that assessed separated NiTi rotary and reciprocating instruments.

**Design and Methods:**

This review assessed clinical studies involving treatments performed by undergraduate students, graduate students, and endodontic specialists. This review evaluated studies using rotary instruments, reciprocating instruments, and hybrid techniques. The number of uses of the different NiTi rotary and reciprocating systems was also assessed.

**Results:**

The incidence of separation for rotary instruments ranged from 0% to 23%. Rotary instruments were used from 1 to 50 times depending on the instrument and tooth type. The lowest rate of incidence separation for rotary instruments was obtained by undergraduate students, using a hybrid technique. The separation incidence for reciprocating instruments ranged from 0% to 1.71%. Reciprocating instruments were mostly single-used; one study reported their use up to 3 times. Separation rate in reciprocating instruments was similar in single-use or in multiple uses 0.2%.

**Conclusions:**

Separation of instruments has dropped recently and seems to be a minor problem in current Endodontics. Multiple uses of NiTi rotary instruments are a possibility without significantly increasing the risk of instrument separation. Single and multiple uses of NiTi reciprocating instruments are also associated with low incidence of separation. Attempting to remove separated instruments should be carefully evaluated.

## 1 INTRODUCTION

The introduction of the Nickel-Titanium (NiTi) alloy in endodontics has rendered the manufacturing of more flexible and resistant instruments [1]. Since the initial hand files, the use of this alloy has permitted centralized and reliable preparation [2]. Currently, NiTi rotary instruments are part of the daily armamentarium of the endodontist and general practitioner with a great variety of instruments presenting different cross-sections, tapers, and new variations of the original alloy [3].

Up to the present moment, due to the anatomic complexity, none of the systems has been able to a complete cleaning of the root canal system [4]. However, NiTi rotary and reciprocating instrum of debris, while maintaining the centralized preparation [5, 6]. The possibility of dentinal defects creation has been suggested [7]; however, recent studies have demonstrated that dentinal defects are unlikely to be related with root canal instrumentation [8]. Despite the advantages of NiTi instruments, the risk of instrument separation is still a concern for the practitioner [9], mainly because this event might occur without previous sign of instrument distortion [10].

The incidence of serious incidents involving separated instruments can be considered very low [11]. Panitvisai *et al.*, [9], have also shown that when an instrument remains inside the canal, it is not necessarily related to a poor outcome. However, when the instrument prevents the optimal sealing of the apical third, especially in necrotic teeth, the outcome may be compromised [12]. One important factor to be considered is the risk involved in the attempt of the removal of the separated instrument.

Several studies have shown the behavior of instruments in laboratory studies [13, 14] and after clinical use [15]. The different kinds of forces that lead to instrument separation, cyclic fatigue, or torsional fatigue have been described in different instruments [16]. Pre-flaring and glide path creation has also been recommended in some systems [17, 18].

Recent clinical studies have described the rate of instrument separation in different NiTi instruments operating in rotary or reciprocating kinematics. These studies have presented controversial results according to the operator's experience, the number of use of the instruments, and the kinematics involved [8, 19, 20].

Thus, the aim of this study is to review the studies that evaluated the rate of separated NiTi rotary and reciprocating instruments in clinical situations.

## 2 CAUSES

When a file rotates inside the root canal, it goes through different forces. In a curve canal, the greater risk is related to the cyclic fatigue; in a narrow canal, the torsional or sheer stress is the greater source of concern.

Irregularities in the instruments might lead to separation due to cyclic fatigue. In the curve portion of the root canal, cracks presented in the instrument suffer compression forces when in the inner part of the curvature, and tension forces in the external portion. The more an instrument rotates inside the canal, the greater number of tension and compression acting in these cracks, therefore increasing the risk of separation [21]. Under a clinical point of view, it is notable that this type of fracture might occur without signs of distortion. Rotary instruments presenting larger sizes require attention when used in curved canals to avoid separation due to cyclic fatigue [16].

When the tip of an instrument binds in a canal and the remnant portion of the instruments rotates, it creates a torsion fatigue. By increasing the cross-section area and the number of threads, the torsional stiffness increases. This results in lessening the risk of separation. The clinician should be aware that in addition to the instrument design applied, the electropolishing used in NiTi instruments diminishes the number of irregularities present and helps decrease the risk of instrument separation [22, 23].

According to Sattapan *et al.*, [24], torsion fatigue occurred in 55.7% of the separated instruments assessed after clinical use. On the other hand, Wei *et al.*, [25], found that, after observing 100 NiTi separated instruments during clinical use, 91% of the separations were due to cyclic fatigue, torsion fatigue occurred in 3% of the cases and, the combination of both in 6% of the cases.

Therefore, the selection of a proper instrument is paramount to avoid separation. It seems reasonable that flexible instruments are recommended for curved canals and stiffer instruments to avoid torsional fracture.

## 3 ROTARY KINEMATICS

The engine-driven instrumentation of root canal system is an aim of clinical endodontics since the beginning of XX Century. These tools aim to decrease the preparation time and simplification of root canal instrumentation. Nonetheless, in the early era of engine-driven instruments there was a high risk of instrument separation when compared to hand instruments. Instrument separation of NiTi Rotary instruments might occur due to torsion, cyclic fatigue or a combination of both forces.

The clinical approach to discard Rotary instruments, aiming to avoid the separation is the number of uses. However, the number of times an instrument can be used with minimum risk of separation is still unclear: different studies show controversial data.

A study has compared cyclic fatigue resistance of ProFile instruments used in maxillary and mandibular molars with a control group of unused instruments [15]. That study showed no difference between the groups, therefore concluding that ProFile instruments can be safely used in up to 4 molars. Cheung *et al.*, [26], have clinically evaluated the separation rate of ProTaper S1 instruments. The instruments were used up to 4 times in molars, 20 times in premolars, and 50 times in incisors and canines; the 325 instruments collected showed a separation rate of 23%. Wolcott *et al.*, [20], found a separation rate of ProTaper instruments of 2.4%, and no difference was found in the incidence of separation for the first 4 uses of the instrument. An evaluation of Lightspeed instruments showed a separation rate of 3.7%; the remarkable result of that study is that 5 out of 6 instruments were used up to 11 times [27].

**Fig. (1):**
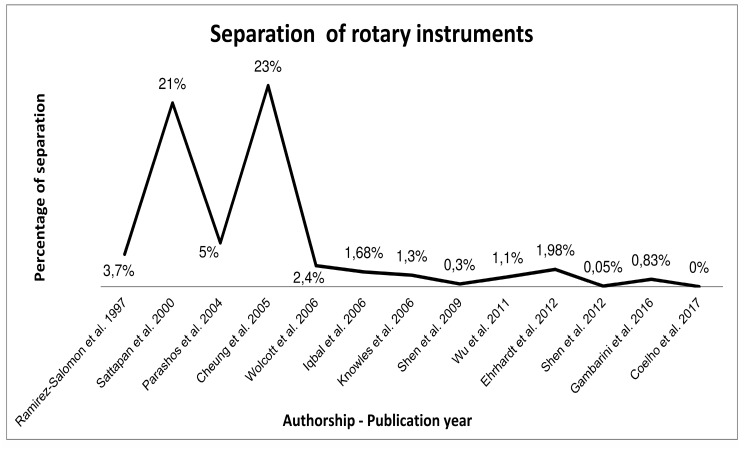
Clinical studies assessing separation of NiTi rotary instruments.

Recent studies, however, have presented lower rates of instrument separation (Fig. **1**). Ehrahdt *et al.*, [28], have evaluated the Mtwo NiTi rotary instruments. After a glide path creation, 1.98% of the files separated; the number of uses in this study was up to 5 times unless the instrument presented signs of distortion. Wu *et al.*, [29], found 1.1% of separated ProTaper instruments even with multiple uses: 3 times for molars, 10 times for premolars, and 30 times for anterior teeth. In that study, the instruments were only discarded after single-use in complex cases. Shen *et al.*, [30, 31], have presented 0.3% for ProFile instrument files used up to 3 times, and 0.05% for ProFile Vortex files used by graduate students in a single use regimen. Coelho *et al.*, [32], have presented no instrument separation in an undergraduate endodontic clinic. The NiTi rotary Vortex instruments were used in 3 root canal treatments if no sign of distortion was present [32]. A recent clinical study has shown a separation rate of 0.83% for the Twisted File Adaptive after the use in 3 molars; no instrument separation occurred in the 2 first uses [33].

The aforementioned findings are in agreement with a previous study. By evaluating 7,159 instruments discarded by 14 endodontists from 4 different countries, Parashos *et al.*, [34], evaluated the factors that might lead to instrument separation. It seems that the number of uses is not a primordial factor that causes instruments to separate. Indeed, multiple uses of instruments are not necessarily related to increase the risk of separation as far as NiTi rotary instruments are properly assessed for signs of distortion or discarded when submitted to complex anatomies. The clinician should be aware that while an instrument can separate without previous unwinding, this deformation suggests that the instrument should be discarded immediately [35].

## 4 RECIPROCATING KINEMATICS

A single-file reciprocating system for NiTi instruments has been suggested, claiming to diminish the necessary steps for root canal preparation. Besides, the single use of these instruments would decrease the risk of cross-contamination and instrument separation [36]. Indeed, laboratorial studies demonstrated that the shift of the kinematics, presenting a cutting angle larger than the relief angle, increases the resistance to the cyclic fatigue when the reciprocating asymmetrical movement is applied [21]. The critical areas of stress move progressively to new locations during the periodical change of the angle, thus distributing effectively the areas of stress to different points of the instrument decreasing the damage and increasing the life span of the instrument.

**Fig. (2):**
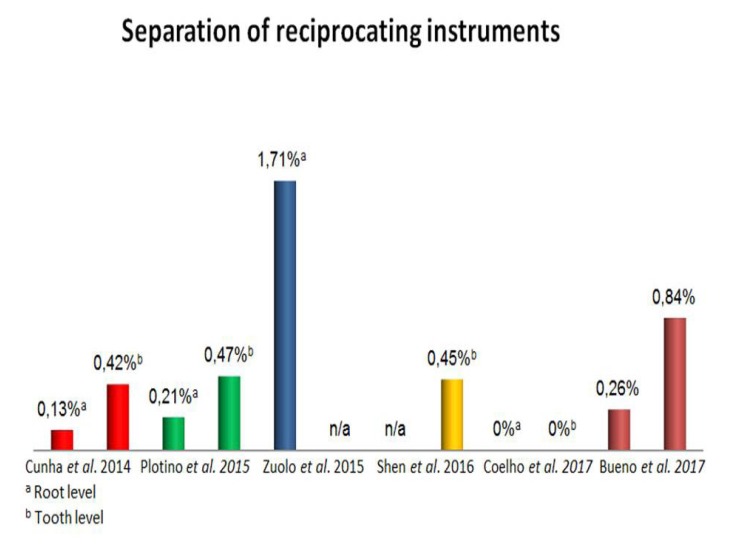
Reciprocating Instruments - percentage of separation in clinical studies. * Only MB2 canals evaluated in this study.

Clinical studies have demonstrated that these instruments present a low rate of separation when a single use approach is applied (Fig. **2**). Cunha *et al.,* assessed 711 cases treated with the WaveOne system; after the creation of a glide path, the instruments were used to instrument 2,215 canals in posterior teeth. The separation rate of this prospective study was of 0.42% on a tooth level and 0.13% considering the number of canals [19]. Shen *et al.*, [37], have presented 0.5% of 438 WaveOne instruments collected after clinical use; Coelho *et al.,* have shown no separation of WaveOne instruments used in an undergraduate endodontic clinic [32]. The WaveOne system is manufactured with an M-Wire NiTi alloy which presents 390% more cyclic fatigue resistance then regular NiTi [38]. A recent study assessed the negotiability of MB2 canals by using the Reciproc R25 instrument, a reciprocating single-file system; within 341 successfully instrumented canals, the separation rate was 1.7% [39]. A clinical evaluation of 3,780 root canal preparations done in primary and retreatment cases with the Reciproc system led to similar results: 0.4% of separation per tooth and 0.2% per canal [40]. Despite the similar alloy and kinematics used in the WaveOne System, Reciproc does not require a glide path. The specific cross-section of Reciproc follows the natural curvature of the root canal, sparing the glide path step.

A recent study has shown that both WaveOne and Reciproc instruments presented low rates - 0.2% of separation even after 3 uses [41]. The single-use recommendation of these instruments in based on concerns in regards to cross-contamination and risk of separation. It is our understanding that whether or not these instruments should be discarded after single use, the same procedure should be applied to hand and rotary instruments. As the kinematics increase the time required to fracture [13], it seems reasonable that, under appropriate case selection and observation after using, the instruments might be used multiple times.

## 5 OPERATOR EXPERIENCE

Knowles *et al.*, [42], assessed 3,543 canals treated in a 24-month interval by undergraduate students. The separation rate found was 1.3% of Lightspeed instruments. Hanni *et al.*, [43], in 87 cases among 40 undergraduate students, showed no file separation; the number of uses in this study is not specified. It is important to emphasize that those undergraduate students had an intense preclinical training, having opportunity to separate instruments without clinical consequences. Shen *et al.,* have presented 0.3% of ProFile Vortex instrumented, separated in a 4-year interval. A following study showed 1 ProFile Vortex instrument separated in 2,203 instruments (0.05%); in both studies, the cases were completed by undergraduate students. A recent study also has shown no instrument separation for NiTi Rotary and reciprocating instruments [32]. The cases were performed by 3^rd^ and 4^th^ year undergraduate students. That study followed the American Association of Endodontists selection case guidelines, meaning that the 715 cases treated were primary treatment of teeth, presenting no complex anatomies.

Iqbal *et al.*, [10], evaluated 10,237 canals treated in 4,685 cases by graduate students in a 4-year interval. Different NiTi rotary systems were used (Light Speed, ProTaper, ProFile, GT Taper, and K3); the overall rate of instrument separation was 1.68% on a tooth level and 0.67% on a root level. Graduate students were assessed by Shen *et al.*, [37], when the WaveOne reciprocating instruments were used. That study showed that single-use of WaveOne instruments separated in 1 out of 85 cases (1.17%) and 1 out of 90 cases (1.11%) in one of the specialist clinics assessed. Three endodontic specialists’ clinics had no separation for the WaveOne system.

Some other studies assessed the separation rate of instruments after clinical use by endodontic specialists. Satappan *et al.*, [44], evaluated the Quantec NiTi rotary instruments collected during a 6-month interval. The separation rate registered was 21%. However, the authors could neither assure that the full sequence of instruments was used nor the number of uses for each instrument. An important aspect of this study is that the instruments were discarded when the cutting efficiency was clinically observed or when signs of distortion were noted. Other studies that evaluated separation rate of rotary NiTi instruments presented better outcomes such as 1.98% for the Mtwo system after a glide path creation [28], 3.7% for Light Speed Instruments [27], 2.4% for ProTaper instruments [29], and 0.83% for Twisted Files Adaptive [33].

Likewise, three recent studies presented similar results with reciprocating instruments used by Endodontic specialists [19, 37, 40]. The rate of instrument separation was low for both WaveOne and Reciproc instruments evaluated in these recent studies. While in the past pre flaring or glide path were recommend to avoid instrument separation [17], the Reciproc system does not require such steps. Yet, the risk of separation of this system is, at least, as low as others. This might be considered an important advantage for clinicians, reducing preparation time and decreasing the risk of separation.

The incidence of separation among undergraduate and graduate students seems to be similar to the ones done by endodontic specialists. This might be due to the case selection adopted (usually primary treatments of uncomplicated cases), the assistance by specialist faculty during the procedures, and the intense preclinical training. General dentists can take advantage of NiTi rotary and reciprocating instruments.

A comparison among different studies is not an easy task; different files were used with different cross-sections and number of uses. For rotary instruments, a reasonable reuse of instruments seems to not increase the risk of instrument separation^34^. Some studies do not state the number of uses of the instruments. However, it is remarkable that the incidence of instrument separation has dropped in the last few years. A better treatment of the instrument´s surface, the novel thermomechanical treatment, kinematics, and case selection might have contributed in obtaining these results.

## 6 INSTRUMENT REMOVAL

Nevertheless, the clinician should decide the better approach when faced with the situation of an instrument separation. Removal of the separated portion, by-passing the instrument, or just not attempting to remove it should be considered. It is worthwhile to notice that instrument separation is not directly associated to failure. A follow-up of 8 patients with irretrievable instruments has shown that after 5 years, 100% of these patients presented functional teeth [45]. Only 12.5% of these patients presented with radiographic characteristics of no healing. That is likely to happen if the fragment prevents a proper cleaning of the apical third in a necrotic case [9].

A classical instrument removal is the Masserann kit, which has been shown to remove up to 55% of the instruments. The major drawback of this system is that the instrument is removed at the expense of dentin destruction [46]. Nevares *et al.*, [47], have demonstrated in a clinical situation that the combination of D.O.M and ultrasonic tips are efficient for the removal of separated instruments. In that study, 85.3% of the visible fragments were removed or by-passed when visible, and 47.7% removed or by-passed when not visible. Similarly, a previous study has shown that 83.33% of the instruments could be by-passed [27]. Usually, by-passing the instrument is the first step in instrument removal procedures. If this step is achieved, the outcome is not different from removed instruments, so the separated instrument management can be considered successful [48].

Gentle Wave is a new approach for removal of separated instruments [49]. It has promoted in vitro the removal of 42% to 91% of the fragments, depending on the curvature and location. However, this information is still limited to a laboratory study and with fragments of 2.5mm, which is not the most frequent in a clinical situation. Therefore this *in vitro* study might have overestimated the actual benefits of applying this device *in vivo* for separated instruments removal.

It is our understanding that an attempt of instrument removal after the curvature is too risky to overtake the benefits. The data of previous studies is still controversial in regards of the benefits of instrument removal. Meanwhile several manufactures advertise their products which might be assessed with caution. A higher benefit would be achieved by following-up with the patient clinically and radiographically, especially in cases of vital pulp. Periapical microsurgery should be the best solution for these situations in most cases.

## CONCLUSION

Separation rate of NiTi rotary instruments has dropped recently and seems to be a minor problem in current Endodontics. The number of uses of an instrument with minimum risk of separation is still unknown. General dentists can benefit from the use of NiTi instruments as soon as the case is properly selected. Attempting to remove separated instruments should be carefully evaluated, as they can be often by-passed or be kept inside the root canal without compromising the outcomes.
